# Lessons Learned: Beta-Testing the Digital Health Checklist for Researchers Prompts a Call to Action by Behavioral Scientists

**DOI:** 10.2196/25414

**Published:** 2021-12-22

**Authors:** Rebecca Bartlett Ellis, Julie Wright, Lisa Soederberg Miller, Danielle Jake-Schoffman, Eric B Hekler, Carly M Goldstein, Danielle Arigo, Camille Nebeker

**Affiliations:** 1 School of Nursing Indiana University Indianapolis, IN United States; 2 Exercise and Health Sciences University of Massachusetts, Boston Boston, MA United States; 3 Department of Human Ecology University of California, Davis Davis, CA United States; 4 Department of Health Education and Behavior University of Florida Gainesville, FL United States; 5 Herbert Wertheim School of Public Health and Longevity Science University of California San Diego La Jolla, CA United States; 6 The Design Lab UC San Diego La Jolla, CA United States; 7 Department of Psychiatry and Human Behavior Warren Alpert Medical School of Brown University Providence, RI United States; 8 Department of Psychology Rowan University Glassboro, NJ United States

**Keywords:** digital health, mHealth, research ethics, institutional review board, IRB, behavioral medicine, wearable sensors, social media, bioethics, data management, usability, privacy, access, risks and benefits, mobile phone

## Abstract

Digital technologies offer unique opportunities for health research. For example, Twitter posts can support public health surveillance to identify outbreaks (eg, influenza and COVID-19), and a wearable fitness tracker can provide real-time data collection to assess the effectiveness of a behavior change intervention. With these opportunities, it is necessary to consider the potential risks and benefits to research participants when using digital tools or strategies. Researchers need to be involved in the risk assessment process, as many tools in the marketplace (eg, wellness apps, fitness sensors) are underregulated. However, there is little guidance to assist researchers and institutional review boards in their evaluation of digital tools for research purposes. To address this gap, the Digital Health Checklist for Researchers (DHC-R) was developed as a decision support tool. A participatory research approach involving a group of behavioral scientists was used to inform DHC-R development. Scientists beta-tested the checklist by retrospectively evaluating the technologies they had chosen for use in their research. This paper describes the lessons learned because of their involvement in the beta-testing process and concludes with recommendations for how the DHC-R could be useful for a variety of digital health stakeholders. Recommendations focus on future research and policy development to support research ethics, including the development of best practices to advance safe and responsible digital health research.

## Introduction

### Background

The increasingly familiar term *digital health* was defined by a United States National Institute of Mental Health working group in 2017, as the *“*blending of mobile health (mHealth) and health information technology (smartphones, wearable sensors, web-based resources, and electronic health records) with genetic, biological, social, and behavioral science to help consumers, clinicians, and researchers measure, manage, and improve health and productivity” [1]. For this commentary, we focus on commercially available and research-grade digital health strategies used in behavioral health research and reflect on the ethical, regulatory, and social behavioral issues inherent in this work.

The ability to leverage digital health strategies and tools (eg, wearable and remote sensors, social media platforms, and mobile apps) to support health research holds enormous potential for behavioral and social scientists by offering accessible, scalable, and cost-effective approaches to delivering interventions to promote health behavior change, prevent disease, identify illness, and facilitate diagnosis [2]. The US National Institutes of Health began funding digital health research over a decade ago and, although the use of digital tools and strategies in research remains somewhat novel, it is escalating rapidly [3,4]. A study of United States National Institutes of Health funding of digital health research documented a 386% increase in funding of digital research between 2005 and 2015 [4]. Digital tools and strategies are increasingly used to reach populations previously understudied in biomedical research [3,5,6], including vulnerable populations with stigmatizing illnesses [7]. In the United States, the rapid onset of the pervasive technology era has preceded the development of ethical guidelines and regulatory infrastructure, which leaves researchers and potentially patients or participants vulnerable in making decisions about the selection of digital technologies and decisions about whether to participate in research. Although efforts are moving forward, these regulatory and governance gaps challenge our scientific community to inform responsible practices in digital health research, particularly new challenges and *unknown unknowns* with regards to risk assessment and data management [8,9].

Harnessing these technologies for use in health research comes with new social and ethical responsibilities to ensure that the products are effective, accessible, usable, sustainable, considerate of privacy expectations, and with good faith efforts to secure volumes of personal and sensitive health information—all of which influence the potential risks of harm and potential benefits of using such technologies [10]. The ethical responsibility to evaluate digital products for use in health research lies not only with those developing the technologies for research-grade use but also with companies that sell these products. Moreover, other key stakeholders, including researchers using digital tools and strategies to study health promotion and ethics review boards (eg, institutional review board [IRB] in the United States, research ethics board in Canada and research ethics committee [REC] in the European Union) that are charged with protecting research participants, have an important role in shaping ethical digital health research [9,11]. However, for researchers, digital health guidance for selecting tools and strategies is lacking. To promote informed decision-making in keeping with social and ethical responsibilities of those who conduct research, the Digital Health Checklist for Researchers (DHC-R) was developed [10]. This paper describes researchers’ perspectives when beta-testing the first iteration of a digital health framework and checklist. Although the perspectives voiced in this paper are those of US-based researchers, we anticipate that our lessons learned, recommendations, and call to action will be relevant globally where regulations and standards to guide ethical digital health research are either limited or nonexistent.

### DHC-R Background

**Figure 1:**
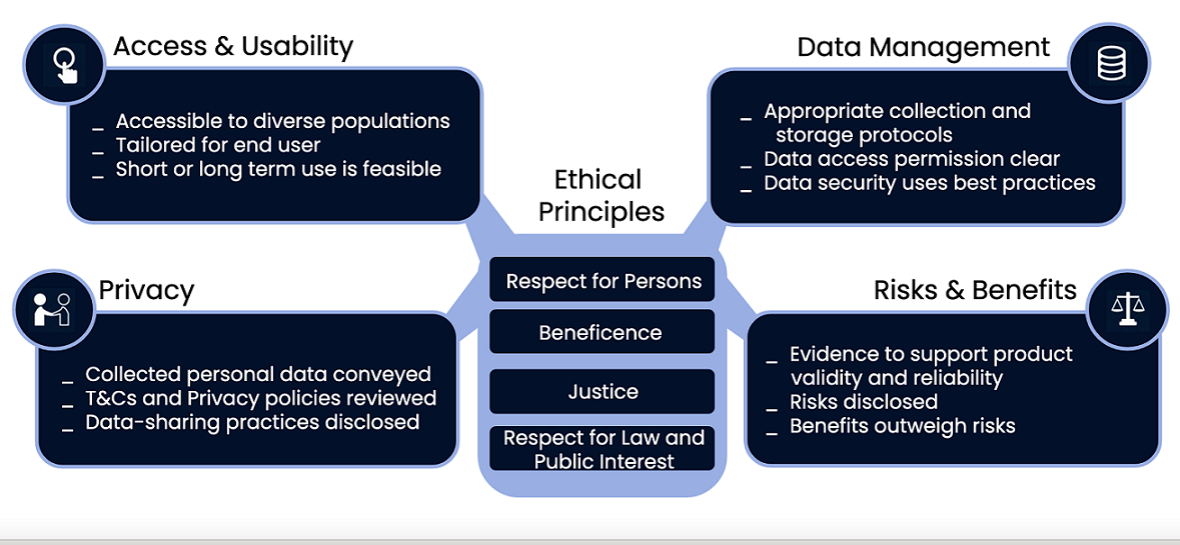
Digital health framework with examples of checklist prompts embedded within each domain (used with permission of C. Nebeker, ReCODE Health).

The DHC-R is grounded in well-established ethical principles of biomedical and behavioral research and supports the following 4 domains: (1) access and usability, (2) privacy, (3) data management, and (4) risks and benefits (Figure 1) [12]. The ethical principles of respect for persons, beneficence, justice [12], and respect for law and public interest form the core of the DHC-R framework (Figure 1). The DHC-R is licensed under a Creative Commons Attribution-Non-Commercial 4.0 International License (2018-2020) and available through the Research Center for Optimal Digital Ethics (ReCODE) Health research tools webpage [13].

These 4 domains intersect with foundational ethical principles that undergird responsible practices in health research. However, researchers face a lack of consistent governance in digital health spaces. Regulations such as the US Health Information Portability and Accountability Act (HIPAA) do not always protect the granularity and volume of data and the safe storage of data derived from digital tools and strategies [14]. Moreover, not all entities conducting digital health research are governed by federal regulations created to protect research participants. This inconsistent governance creates confusion about how to assess risks of harm, manage data, and convey information to potential participants [15]. Awareness of these inconsistencies is useful when making choices about which digital solutions may work best to support the research aims and be safe for use with research participants.

### Beta-Testing Approach

The DHC-R checklist was developed in 2 phases and has been reported elsewhere [10]. The DHC-R was developed in the United States and anchored to ethical principles that guide biomedical and behavioral research in the United States. Briefly, during phase 1, an expert panel was convened to review a decision-making framework published by the American Psychiatric Association, which was designed for clinicians who might prescribe or recommend a mobile app to their patients [16]. Phase 2 involved beta-testing a modified version of the American Psychiatric Association checklist for use by digital health researchers. Beta-testing involved a group of behavioral scientists who agreed to serve as the second expert panel. Expert panel members were asked to identify a recent digital health research study that they had designed and obtained IRB approval to conduct. Each panel member then applied the modified checklist, administered via the SurveyMonkey (Momentive Inc) platform, to their specific use-case study. Following completion of each domain area within the checklist (eg, privacy and data management), each panelist was asked to comment on the clarity of the prompts and whether additional criteria or content should be included (eg, *is anything missing* or *anything else to add*).

When beta-testing was completed, the lead authors (RBE and CN) reviewed all qualitative and quantitative responses and subsequently revised and published the DHC-R development process. Qualitative comments also revealed personal reflections by the panelists and a few concerns; specifically, that their prospective risk assessment may have been incomplete. With the goal of exploring their experiences during the beta-testing process, the lead authors scheduled 2 meetings with the panelists. After the first meeting, the lead authors reviewed open-ended comments and meeting notes and identified emerging themes using a thematic analysis approach [17]. During a second meeting to confirm and refine the themes, the lead authors discussed how best to share these valuable and personal insights leading to this paper’s *lessons learned* summary. For context, the lessons learned presented in this paper arise from the authors’ experiences working with technologies and research participants in the United States. Collectively, we conducted behavioral change intervention research that is both preventive (ie, physical activity and healthy eating) and aimed at treating chronic conditions, such as kidney disease, diabetes, and cardiovascular disease. We leverage technologies, such as wearable sensors, mobile apps, web-based social networks, and other connected technologies (ie, smart pillboxes and weight scales) in our research. Therefore, the lessons learned presented in this paper are shaped based on the United States context, including relevant United States legal protections of human subjects in research, and other relevant legislations such as US privacy laws. The goal of sharing our collective experience as panel members and behavioral scientists is to support the broader digital health research community and encourage other stakeholders to share responsibility for individually and collectively shaping ethical and responsible practices.

## Results and Lessons Learned

The following 3 key themes were labeled: (1) researcher vulnerability, (2) lack of control, and (3) researchers’ responsibility for human research protection. For each theme, we address what actions can be taken and future directions to consider.

### Researcher Vulnerability

#### Overview

Reflecting on the design and implementation of their study use case left many panelists feeling vulnerable. This vulnerability stemmed from recognizing, in our role as behavioral scientists and researchers, we lacked a framework to guide the selection of the digital health tools and strategies used in our research studies. Without guidance, we needed to rely on our personal experience, technical savviness, and best judgment. Before beta-testing the DHC-R, there were no evidence-based tools or resources available to inform the selection of a digital health tools or strategy that would be appropriate for our studies and safe for participants. As researchers, we have used important and practical considerations for selecting a digital health product, such as whether the technology could be adapted to meet the needs of the research study across research sites and whether it had reasonable technical specifications. However, too often, we were not aware of important ethical aspects, and although our studies had received IRB approval, there were aspects of the digital tools and strategies we were not familiar with or knew how to consider prospectively when planning our research. Often, these issues are not known until a problem arises, which is what the DHC-R is working to preempt by helping researchers develop awareness as well as decision-making skills needed to be purposeful in advancing responsible and safe digital health research. The following response to the DHC-R beta-testing survey conveys this vulnerability:

Yes [I would do things differently]...That said, I also feel powerless as there is so little, I feel I understand in making decisions on use.

#### Reducing Vulnerability

As researchers, we are trained to ask scientific questions and design studies to answer these questions. We have become experts in our respective disciplines; however, when we have the opportunity to use new tools or methods, we must recognize that we are novices and seek guidance from those with greater expertise, as well as diverse experiences. We may find that working with a technologist, privacy expert, or participants can help to shed light on the *known unknowns* and potential *unknown unknowns* that are inherent when learning how to use a new research tool. Recognizing our vulnerability is necessary and humbling and will certainly lead us to become better scientists. Nevertheless, it is important to balance the fear of making the wrong decision with that of moving fast and potentially causing damage and harm. The slogan *“*Move purposefully and fix things” aligns with our intention of embracing vulnerability [18].

#### Future Directions

Our developmental research revealed that using the initial checklist can facilitate reflection on factors that influence responsible digital health research [10]. Whether this reflection process prevents one from blindly moving into work that could be risky is unknown. We also do not know if reflecting on potential risk may discourage one from pursuing important health research. Although additional research on the DHC-R is required, the checklist was useful in prompting our awareness of important considerations when selecting digital tools and strategies for health research.

The framework (Figure 1) may reduce the vulnerability. Researchers are encouraged to consider digital tools and strategies from both the researcher and participant perspectives during the study design phase. The domain of *access and usability* prompts the consideration of the extent to which training may be required. For example, although commercial health wearables (eg, Fitbit) are designed for ease of use, participants may not intuitively know how to use real time data to make decisions about their future health behaviors. Assessing the need for and potential methods to train participants could prevent technology naivete from limiting intervention effects and influencing outcomes.

The *privacy* domain prompts researchers to take a deep dive into the vendor’s terms and conditions of service to understand what data are collected, where data are stored, and how data might be shared. For example, researchers should be able to select specific variables of interest from a health technology platform (eg, location and time of day) without burdening participants to provide these data. Moreover, requiring participants to download the data file and transfer it to researchers could compromise the data quality and security. Assessing the need for a third-party data management system is also an important consideration.

**Figure 2:**
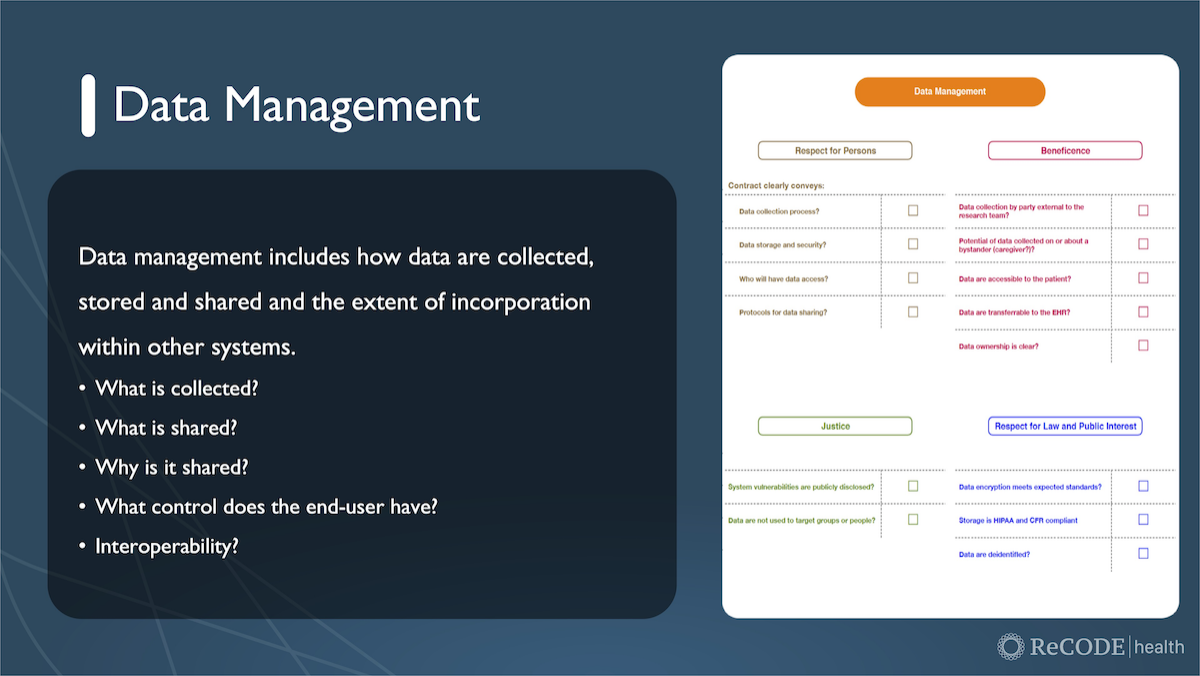
Example of data management domain from the digital health checklist (used with permission of C. Nebeker, ReCODE Health).

Finally, items in the *risks and benefits* domain of the checklist cue researchers to think about what evidence exists to support using the technology (reliability) and the validity of the device for the particular study in question. Checklist prompts (Figure 2) guide the researcher to consider potential risks and weigh them against the potential benefits of the technology.

During beta-testing, one comment describes how future thinking may be demonstrated: “I plan to more fully consider what details to include in the informed consent, particularly about data sharing.” Applying the checklist used during beta-testing was beneficial in that it prompted reflection. Moving forward, we anticipate that other researchers will find it useful to prospectively consider how a particular digital health tool or strategy aligns with their goals of doing good science and protecting research participants. For example, a researcher evaluates an app that also happens to collect GPS data, but GPS data are not needed for the research study. In this case, the DHC-R would prompt the researcher to think about either using another app, if the GPS feature cannot be *turned off*, or explaining to participants during informed consent that these data are being collected so that they can make an informed decision about whether to volunteer. Technologies on the horizon, such as the *smart toilet*, will likely continue to challenge our ability to evaluate situations in which researchers collect data that extend beyond their original research questions. The DHC-R may prove useful for decision support today, and in the future, provided it is a dynamic tool that changes with time. These choice points become particularly important if the researcher uses a third-party app that provides little or no control over the use of data outside the research study.

### Lack of Control

#### Overview

Experiencing vulnerability stems from the awareness that researchers sometimes lack control when selecting digital technologies. For instance, researchers often sacrifice control for the benefits of using commercial products. Many commercial products make the application programming interface available to all or select approved researchers. This allows researchers to access data and deploy interventions using commercial products. For example, in a statewide dissemination of a web-based behavioral weight loss program offered through primary care clinics, participants have the option of linking a commercially available calorie counter smartphone app to their patient portal, where they are responsible for uploading weekly data on calories consumed per day, daily exercise minutes, and daily body weight [19]. Calorie counter apps are widely available, but the majority of the most popular ones do not have a researcher-accessible application programming interface. Consequently, researchers are limited in which apps they can use if they wish to access these data. At the same time, smartphone apps regularly add new (or discontinue) features, which may impact researcher access to data or make a training guide for participants obsolete when features change. In the traditional research timeline, commercial digital health products will update repeatedly during the course of a single trial, and these updates are out of the researcher’s control. These updates can wreak havoc with regards to measurement and implementation and undermine the entire study design. Finally, researchers often lack control when they choose to use a commercially available digital tool or strategy because they cannot control modifications made to the data structure, algorithms, or equations, nor control whether the company stays in business, which is crucial to maintain consistent technology support and potentially data management.

Although digital tools and strategies create opportunities for researchers, the field is dynamic, making it difficult to foresee all potential risks. However, we learned that the decision-making domains of the DHC-R helped to focus panelist attention on key areas, including privacy, data management, risks and benefits, access, and usability. In this sense, the DHC-R builds awareness around important decision-making domains. The following comment made during beta-testing reflects this increased awareness:

There were various factors around interoperability and privacy that I did not previously consider, especially with the use of commercial apps within my study. Data from these apps weren’t collected for my study, but I did request that participants try a variety of apps, which could have put them at risk for loss of confidentiality that wasn’t as clear at the time of the study. Will consider this more in the future and also include transparent language in the consent forms.

#### Future Directions

To move the field and researchers toward building awareness of the controllability of the digital tools and strategies, the DHC-R is best used during the planning phase of research. Not only can it be useful in prompting researchers to consider what they can and cannot control and guide development of contingency plans, the DHC-R can be used throughout the project for ongoing assessment of factors that influence responsible and ethical practices. By conducting an ongoing assessment of the products selected for a particular study, we anticipate an increased sense of control that comes with being informed. In addition to being informed, we as individuals and members of the digital health research community need to be honorable stewards of funding received to conduct behavioral health research with integrity. The responsibility to take action resides with all in the digital health research ecosystem; however, it is not presently a norm. As such, we collectively need to commit to affecting changes in standards such that poor practices do not become the default standards. For example, if a product is not *research* friendly (eg, terms of use conflict with federal regulations for human subject protections and platform changes without notification), then those vendors become less desirable than vendors that are supportive to the research enterprise. Moreover, there is a need to prioritize the training of researchers in the pipeline such that they recognize their role in shaping policy and the dynamic development of best practices. With these added responsibilities, we recognize that research funders need to step up to support research on research ethics in the digital health sector, ideally as part of strategic planning. These collective efforts will serve to advance the development of future digital health research and raise awareness of what is acceptable verses a deal breaker.

### Researcher Responsibility in Protecting Participants

#### Overview

Two important lessons emerged about the researcher’s responsibility. The first lesson is recognizing our responsibility for due diligence in the protection of human subjects when selecting technologies and embedding them in our study design. This includes how researchers communicate and plan studies that are submitted to the IRB/REC. The second lesson is on how we take our DHC-R assessment outcome and communicate it in a way that potential participants can understand and make an informed decision about whether to participate. These 2 lessons highlight researcher responsibilities and suggest actions needed when planning future studies and how the DHC-R can assist with research design and implementation.

#### Due Diligence

Using the DHC-R places a shared responsibility of due diligence on both the researcher and IRB/REC—rather than relying solely on the IRB/REC or other body to determine participant safety. For example, if researchers use the DHC-R during the protocol development phase and then share that evaluation process with IRB/REC members, researchers demonstrate that they are thinking critically about designing research with prioritized participant safety. However, there were concerns that an IRB/REC may use additional knowledge about digital health tools and strategies to act more conservatively and overprotect participants, thus slowing the approval process and, possibly, stopping important health research from occurring. Moving forward, it is critical that both researchers and review boards play an equally active role in developing awareness of factors that influence study risks and benefits and learn how to use decision support tools such as the DHC-R.

We collectively agreed that researchers should not exclusively outsource the responsibility of risk assessment to the IRB/REC. Specifically, researchers should not rely on the review board as an *ethics* expert, especially in the area of rapidly changing tools and strategies used in digital health research [8]. For example, digital technology companies can be acquired by other companies, which is not often discussed in typical research consent forms. In such cases, researchers must consider how the acquisition affects research data collection, storage, and sharing while considering the long-term availability of the product for continued use. Researchers should plan to communicate this information to prospective participants during the consent process and provide updates to the currently enrolled research participants. In effect, the DHC-R helps to shape the research protocol with respect to complex aspects of data management, privacy, access, and usability, and also how to convey these concepts when crafting the information conveyed during the informed consent process.

#### Moving From Responsibility to Accountability

The DHC-R can help researchers identify ethical and regulatory issues that have typically been left to the review board members to identify and resolve. Moving forward, researchers as well as others who make up the digital health research sector must design an ecosystem where we collectively use checks and balances. The organization of this ecosystem is beyond the scope of this paper; however, we highlight the following steps. First, to improve informed consent, we need to make information accessible and meaningful and to present it in a way that facilitates sound decision-making. At present, researchers tend to use words to convey complex concepts that may not be useful to people with low technology and data literacy. Designing a consent process that actually informs decision-making may require that researchers partner with prospective participants and communication and design experts to learn how best to convey information. This goal is not trivial and will require dedicated funding to overhaul how we approach informed consent. For now, the DHC-R provides a framework for deep consideration of what information participants may need to make informed decisions. In addition, the DHC-R facilitates an opportunity to consider how researchers can best assist individuals in becoming better informed with respect to information contained in a terms of service agreement. Although many agree that service terms and privacy agreements are not meant to be read or understood, as researchers, we have an obligation to educate prospective participants and help them to understand what permissions are given when accepting terms to use a commercial product (eg, Fitbit and Facebook) [20]. This education can include information on privacy limitations and data management, along with the potential unknowns inherent in digital health research participation.

#### Future Directions

When technology is used in health research, data security and privacy responsibilities should be shared to an extent by all stakeholders, including technology developers, researchers, hosts, and participants. It is also the researcher’s responsibility to inform participants when product functionality that may alter privacy and data management risks changes. Presently, institutions focus on research risk assessments to reduce legal culpability. That may be important; however, the need for institutions to help researchers to develop sound data management protocols that protect research data is critical, particularly when potentially granular personal health data collected using digital tools and strategies are not covered by United States HIPAA regulations. One beta-tester indicated the following:

I think it was critical that when using a commercial device, we included the creator [tech developer] as a co-investigator. This helped us think through generalizability and future applicability issues once the software and hardware are updated. I have done other studies in the past where we did *not* do this, and it was a mess. I highly recommend doing this whenever possible.

Moving forward, we envision the DHC-R inspiring awareness of our collective responsibility in framing the ethical, legal, and policy-related decisions that support a robust digital health research culture. Ideally, researchers will be better able to identify problems and craft risk management solutions that align with developing best practices—this includes elevating awareness of products that are neither *research friendly* nor in the best interest participants who engage in our research studies.

## Discussion

As researchers, we have a responsibility to (1) lead the narrative for accountable, fair, and transparent practices; (2) inform best practices by sharing experiences and conducting empirical research; and (3) help our colleagues avoid pitfalls. Following our experiences of beta-testing the checklist, which led to the DHC-R, we realized that by openly sharing our experiences, both failures and successes, we could advance the development of best practices. The DHC-R is a new decision support tool that uses a framework consisting of the following 4 domains: (1) access and usability, (2) privacy, (3) data management, and (4) risks and benefits anchored to the ethical principles of biomedical and behavioral research used in the United States [12]. Our lessons learned serve as the starting point for openly sharing successes and failures in selecting and using technologies in health research. However, with the rapidly changing digital landscape, there is an urgent need to engage researchers and the extended digital health research community in the open sharing of experiences to expand the body of lessons learned. The DHC-R provides a structure that could be useful for other responsible parties, including technology developers and IRB/REC members. Scientific and clinical progress is most likely to be achieved if the digital health research community collaborates with global stakeholders and regulatory bodies to ensure safe and innovative science.

To inform best practices, a global and multi-stakeholder perspective is needed, including traditional researchers at academic institutions, health technology companies, and citizen science initiatives. With this diverse research ecosystem comes variation in formal research training, ethics acculturation, and regulations, all of which call for elevating opportunities for engagement and dialogue [15]. Furthermore, the DHC-R was developed and beta-tested with US-based researchers; thus, the perspectives described herein may only be applicable to the United States. Engaging international stakeholders is needed to understand the extent to which the findings are applicable outside the United States.

In considering the *lessons learned*, a cluster of issues emerged surrounding the importance of, and the need for, educating stakeholders, particularly IRB/REC members and participants (who may also be patients). With that in mind, we propose *a call to action* inviting key digital health stakeholders, including review boards, participants, professional societies, and funders, to consider their respective roles in designing a responsible and ethical digital health research ecosystem.

### Ethical and Regulatory Review

The IRB/RECs perform an important function in the review and approval of regulated research with human subjects. However, as technology changes and digital health studies increase, we see gaps in the ability of review boards to be well informed and make appropriate decisions [8]. The digital health community needs to collaboratively develop processes to advance ethical digital health research. Organizations that support IRB/REC professionals (eg, Public Responsibility in Medicine and Research) and recognize the unique challenges introduced by technologies in health research (eg, Society of Behavioral Medicine, American Psychological Association, American Medical Association, and Computer and Human Interaction) are well-positioned to increase educational opportunities specific to the ethical, legal, and social implications of digital health. Over the past decade, we have seen an increase in meetings that bring together experts from across sectors and disciplines, and given the increase in internet-based meetings owing to the COVID-19 pandemic, convening across professions and sectors is easier than ever. However, more work is needed to advance safe, responsible, and ethical digital health research. Researchers also have a responsibility to suggest innovative strategies for protecting patient privacy and safety to their colleagues, review boards, and professional societies. Ethical practices must be shaped as a system rather than being siloed within research areas and organizations.

### Educating Participants or Patients

Educating participants about digital health research has ethical, legal, and practical considerations. From an ethical perspective, it is important to anticipate or consider how to make the informed consent process accessible, especially in studies that are completely remote. There has been some research on the process and use of e-consent [21] and efforts to improve how complex concepts are conveyed through a smartphone or tablet. We encourage efforts to involve participants (and their caregivers where appropriate) as partners and working with prospective participants to complete the DHC-R can be an approach to engage them in study planning, informing study feasibility, and assisting with risk assessment and mitigation planning.

### Professional Societies

We encourage professional societies to foster a culture among members that put the ethical, legal, and social implication responsibilities of researchers at the forefront. To do so, we recommend that professional societies use the DHC-R framework and companion checklist as a decision support tool for member education efforts that encourage the conduct of socially and ethically responsible research. Furthermore, the intersection between academic researchers and industry calls for scientists to become collaborators with technologists, rather than only being seen as end users. To accomplish this, professional societies can encourage industry partners to attend scientific meetings, not solely as vendors or industry sponsors, but as attendees. The DHC-R could be a useful framework for developing targeted education and introducing concepts of ethical decision-making in the digital health sector.

### Funding and Policy

The current funding paradigm, both within the United States and abroad, typically requires compliance with regulatory mandates for human subject protection, including guidance for obtaining and documenting informed consent. However, our existing methods for obtaining informed consent do not require the assessment of the extent to which an approved consent process results in an informed participant; hence, further research is needed here. Funders can also support researchers interested in shaping digital health research policies and norms by creating a mechanism to support the empirical research that will advance evidence-based policy development. Moreover, similar to the importance of having a biostatistician on the research team, researchers should consider engaging a health technology ethicist as a coinvestigator and include aims that advance knowledge of risk assessment and meaningful consent.

### Learning Communities

Learning communities that support safe and open sharing are recommended. As digital health and related new technologies continue to emerge, unknowns are expected and will continue to surface. These unknowns, when they do occur, create learning opportunities from which researchers and other stakeholders could benefit—particularly if we embrace a learning community approach. To accomplish this type of open sharing culture, a platform is required. One such platform that could serve as a model (or be the *go to* resource) is the Connected and Open Research Ethics (CORE) platform hosted by ReCODE Health [22]. To succeed, open sharing would be rewarded. For example, a novice digital health researcher who plans to use wearable sensor technologies to observe free-living daily behavior with the goal of documenting physical activity and then deploying a behavioral intervention may benefit by accessing IRB/REC-approved protocols that have been shared by a more experienced researcher. This can occur at the CORE Resource Library, where IRB/REC-approved protocols and consent documents are tagged and freely shared by and with members of the global CORE network [23,24]. Experienced researchers who share their digital health protocol to help others may be motivated through altruism or with service credits that contribute to productivity metrics for promotion. The motivation to participate as a member of the CORE should be driven by a commitment to advocate and a desire to contribute to the shaping of best practices. How that is realized will be up to our collective community efforts. Alternative metrics or rewards for researcher transparency, whereby researchers get credit for open sharing, will be how researchers are able to lead within the academic community.

### Limitations

The lessons learned and call to action recommendations described in this paper are based on experiences in the United States and framed on current relevant and applicable laws, or lack thereof. Although the United States lacks a regulatory framework to guide researchers in using digital technologies in research, there are emerging state efforts that show promise. For example, in California, the recent privacy regulation will protect resident personal information collected by unregulated, for-profit businesses in cases where the United States HIPAA does not apply [25]. Internationally, there have been advances in privacy and data management. The World Health Organization has published a resolution on health technology assessments to improve decision-making and policy development [26]. In Europe, policy-driven [27,28] impact assessments have advanced from privacy impact assessments [29,30] to mandated data protection impact assessments, which are required when processing data “is likely to result in result in a high risk to the rights and freedoms of natural persons” (General Data Protection Regulation) [28,31]. The United States could learn from these efforts occurring in other countries and align with these best practices, as applicable to the United States. There is much we can learn from other countries that have been successful or are leading successful efforts in these areas.

### Conclusions

Digital technologies offer potential benefits for advancing health research, yet introduce unique ethical, legal, and social implications. For researchers, due diligence in selecting technologies for research purposes requires awareness of known risks and anticipation of unknown risks to both individuals and society. Decision support tools can help researchers systematically consider the selection and use of technologies for use in health research. The DHC-R described in this commentary is one decision support tool that prompts researchers to anticipate participant privacy, consider risk of possible harms against benefits, evaluate access and usability, and review data management protocols. For researchers, the DHC-R can be used to facilitate informed research planning and preempt downstream problems associated with digital health research. Researchers who tested the DHC-R learned valuable lessons, which were synthesized to inform recommendations and a call to action directed at key stakeholders. Encouraging strategies that promote open and transparent discussions about the promise and pitfalls of digital health are required to ensure ethical and trustworthy research. Through collective action to identify issues and unmet needs within the global digital health ecosystem, an ethical and responsible digital health architecture can be realized, but only if stakeholders act now to do their part.

## Abbreviations

CORE: Connected and Open Research Ethics
DHC-R: Digital Health Checklist for Researchers
HIPAA: Health Information Portability and Accountability Act
IRB: institutional review board
REC: research ethics committee
ReCODE: Research Center for Optimal Digital Ethics
